# Beam Tilt Aberration Detection of the Seven-Unit Phased Fiber Laser Array

**DOI:** 10.3390/mi16010038

**Published:** 2024-12-30

**Authors:** Xin Yu, Xingyue Wang, Jing Liang, Cai Liu, Xiaolong Ni, Suping Bai, Jiasu Li, Zeping Liu, Lijie Hou

**Affiliations:** 1 School of Optoelectronic Engineering, Changchun University of Science and Technology, Changchun 130022, China; wxy577694165@163.com (X.W.); 19990527240@163.com (C.L.); nxl@cust.edu.cn (X.N.); baisp@126.com (S.B.); 15831135975@163.com (J.L.); 15943651930@163.com (Z.L.); houlijie3887@sina.com (L.H.); 2 Railway Locomotive and Rolling Stock Faculty, Jilin Railway Technology College, Jilin 132299, China; 15652926744@163.com

**Keywords:** laser, adaptive optics, aberration detection

## Abstract

In this paper, we present a method based on the conjugate image principle and micro-nano optics to detect tilt aberrations of a phased fiber laser array system. A co-aperture optics system was adapted to detect the tilt aberrations of a seven-element phased fiber laser array system simultaneously. A Kepler telescope was designed to construct the conjugate relation between the exit pupil of a fiber optic laser array system and a microlens array and also to match the size of the seven beams and the microlens array. The apochromatic theory was used to meet the multispectral (1064 ± 0.3 nm, 1030 ± 0.3 nm, and 633 ± 0.2 nm) detection needs. A far-field detection unit was also designed to evaluate beam quality. When the actual beam was offset by 1 pixel, the beam tilt was about 0.7 µrad. The maximum detection error of the seven-element system was about 7 µrad. It could not only directly detect the beam’s tilt angle but also maintained detection accuracy while reducing the algorithm complexity.

## 1. Introduction

High-power lasers have vast application potential in diverse fields such as industrial manufacturing, defense, and scientific research. As the demand for higher output power and superior beam quality continues to grow, efforts to advance laser technology are increasingly focused on overcoming the limitations imposed by nonlinear and thermal effects. These effects create a theoretical threshold for single-beam laser output power, which constrains further progress [1,2,3]. One promising solution to this challenge is the development of lightweight, modular fiber laser phased arrays that can combine the outputs of multiple laser beams. This approach, known as beam combining, is a key strategy for achieving higher laser power and has garnered significant attention in recent years. Notably, in 2011, C.X. Yu and colleagues at MIT demonstrated the experimental combination of eight fiber amplifiers, resulting in a 50-fold increase in the peak optical intensity of the combined output compared with that of a single beam. Furthermore, the far-field beam quality achieved 1.25 times the diffraction limit [4]. Building on this work, in 2020, researchers at the National University of Defense Technology successfully achieved a coherent beam combining 107 fiber lasers [5], and in 2021, they realized a 20 kW high-output power laser by combining 20 fiber lasers.

In fiber laser synthesis array systems, beam tilt differences are critical factors that affect both the quality and efficiency of the synthesized beam [6,7]. The primary challenge is how to detect these tilt differences and correct wavefront distortions to maintain high laser beam quality [8,9]. Wang Xiong et al. [10] developed a real-time method to detect beam tilt and wavefront distortions by using the far-field bucket power as a performance evaluation function. By processing spot data captured by CCD cameras, they successfully implemented detection and optimization control for a four-channel fiber laser array, but the use of far-field bucket power as the evaluation function limited its application when the tilt difference was large. Huang Zhihong et al. [11] explored various beam synthesis models and simulated the effects of tilt and shift differences on the performance of the synthesized beam. Their results demonstrated that as the number of synthesized subbeams increased, the power in the far-field bucket decreased more significantly with larger tilt angles, revealing a cumulative degradation effect on beam quality as the number of synthesis channels increased. Geng Chao et al. [12,13] investigated the coherent synthesis of two, three, and seven laser beams arranged in linear, triangular, and hexagonal configurations. Their findings showed that the hexagonal arrangement outperformed the other configurations in terms of synthesis efficiency. Therefore, this study chose a hexagonal arrangement pattern. Furthermore, they observed that increasing tilt differences led to a noticeable deterioration in synthesis performance. Peng Yingnan et al. [14] established a seven-channel fiber laser synthesis system and used the second moment of the synthesized spot as an evaluation function for tilt control in array beam coherent synthesis. They successfully measured spot distributions when the standard deviation of tilt differences did not exceed 30 μrad and employed an adaptive optical fiber collimator for calibration. Their study concluded that adaptive optical calibration technology was highly effective for detecting and correcting tilt differences in fiber laser arrays.

In summary, addressing the detection of tilt aberrations in adaptive optics correction technology is an effective approach to enhancing the quality of synthetic array spots. M. A. Vorontsov et al. [15] used a smaller-sized fiber collimator array to replace individual optical elements, reducing system weight while controlling the tilt of the beam’s wavefront phase. However, this configuration required high precision and could only assess the beam tilt indirectly through the uniformity of light intensity, without directly measuring the tilt angle or detection accuracy. In 2023, Wang Chongchong et al. [16] proposed a wavefront measurement method based on a single Shack–Hartmann wavefront sensor capable of handling large-amplitude tip-tilt and distortion measurements. Although this system simplified the structure and enabled detection, its data processing complexity was high, and its efficiency was low, with the root mean square (RMS) value after calibration being only 0.2λ. In the same year, Fan Zou et al. [17] demonstrated optical coherent combining using a tiled-phase fiber laser array with 19 channels of subapertures. However, the system lacked real-time tracking and calibration capabilities, relying instead on manual alignment for rough adjustments. Additionally, each subaperture required separate calibration, complicating operation and increasing costs. To address the challenge of tilt aberration detection in fiber laser synthetic arrays, this paper proposes a new system capable of directly measuring the beam’s tilt angle. This system not only improves real-time monitoring accuracy but also reduces costs. By directly measuring the phase tilt angle, the system requires fewer iterations and offers higher computational efficiency. The design employs a co-axial receiving detection scheme based on conjugate imaging principles, combined with a small-scale segmentation detection method using micro-nano optical devices. This enables the detection of wavefront tilt in a seven-channel, 30 mm diameter fiber laser array, while also assessing beam quality and uniformity. Experimental validation confirmed the system’s effectiveness.

## 2. Principles

**Figure 1:**
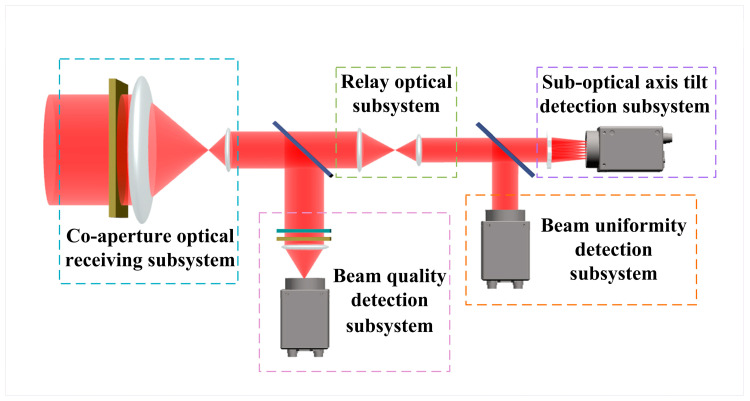
Schematic diagram of beam tilt detection system.

The overall system included a co-aperture optical receiving subsystem, a relay optical subsystem, a suboptical axis tilt detection subsystem, a beam uniformity detection subsystem, and a beam quality detection subsystem. By employing spectral division technology, the light received by the co-aperture optical receiving subsystem was distributed among the various subsystems. This not only enabled large-aperture reception but also made the structure more compact and the volume smaller. During the design process, the components adhered to the following basic principles to ensure the accuracy and rationality of the system design: (1) the co-aperture optical receiving subsystem and relay optical subsystem must satisfy the optical pupil alignment principle; (2) the co-aperture optical receiving subsystem and beam quality detection subsystem must be compatible; and (3) the relay optical subsystem, beam uniformity detection subsystem, and suboptical axis tilt detection subsystem must meet the optical pupil alignment principle. The schematic diagram of the overall system layout is shown in Figure 1.

The primary function of this system was to detect the tilt aberration of laser beams with a diameter of 90 mm and wavelengths of 1064 ± 0.3 nm, 1030 ± 0.3 nm, and 633 ± 0.2 nm, as well as to assess the beam uniformity and quality. The specific wavelength chosen for this study was less affected by reflection, absorption, or scattering when passing through the atmosphere, resulting in a higher transmission rate, which was beneficial for improving space imaging and communication performance. The field of view was set to ±0.25°, adjusted according to the range of the adaptive system’s adjustment units. The co-aperture optical receiving subsystem served as the beam entry for the system, with the entrance pupil diameter set to 90 mm. To accommodate multiple beam splitting of high-energy lasers and weak light detection, the entrance pupil distance was set to 500 mm to allow sufficient space. To ensure that the beam could smoothly enter the subsequent branches after splitting, the optical design included beam splitting and bending. Therefore, the pupil position must be at least 80 mm to meet assembly requirements.

The beam quality detection subsystem took into account the limitations of the detector’s target area, with the minimum target size being 1/2″. According to the formula

(1) $$f_{z}=\frac{Y}{tan\theta },$$

the combined effective focal length of the beam quality detection subsystem should exceed 1000 mm. In the formula, θ represents the field of view angle, *Y* denotes the height of the detector camera’s target surface, and $f_{z}$ is the combined focal length of the beam quality detection subsystem and the co-aperture optical receiving subsystem. Similarly, the effective focal length of the system composed of the suboptical axis tilt detection subsystem, beam uniformity detection subsystem, co-aperture optical receiving subsystem, and relay optical subsystem should also exceed 1000 mm.

According to the equation

(2) $$f_{\gamma }=\frac{f_{z}}{\gamma },$$

the focal length of the beam quality detection subsystem was 166.7 mm. In this equation, γ represents the magnification ratio of the co-aperture optical receiving subsystem, and $f_{\gamma }$ denotes the focal length of the beam quality detection subsystem.

Since the co-aperture optical receiving subsystem and the relay optical subsystem met the pupil alignment principle, the incident beam diameter of the relay optical subsystem was 15 mm. To ensure the integrity of the spot and the accuracy of the calculations, the coverage area needed to be appropriate. A coverage of 600 pixels was sufficient to meet the detection accuracy requirements. The combination of the relay optical subsystem and the co-aperture optical receiving subsystem reduced the beam by a factor of 15, decreasing the beam diameter to 6 mm. Distortion was a crucial factor affecting the accuracy of optical measurements, referring to the degree of image distortion produced by the optical system [18]. When lens distortion was below 1%, the image deformation was not easily noticeable and had a minimal impact on detection accuracy. The specific technical specifications of the system are detailed in Table 1.

## 3. Analysis and Design of the Optical System

**Figure 2:**
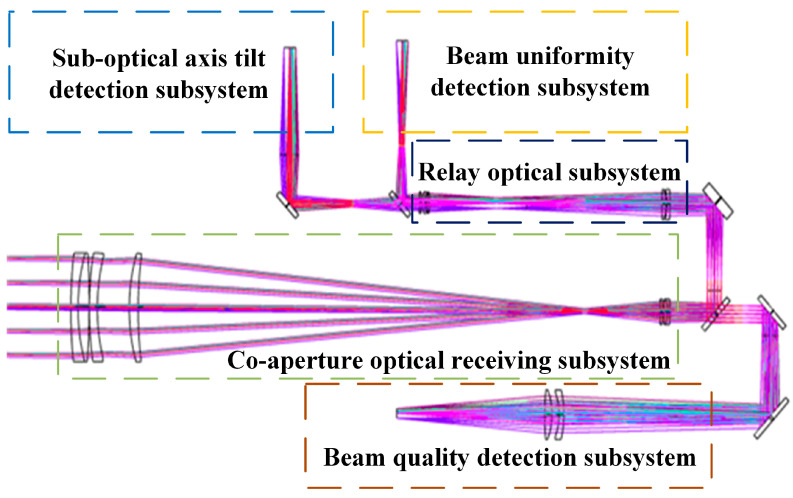
Schematic diagram of the beam tilt aberration detection system.

Figure 2 is the structural diagram of the measurement system. It consisted of the co-aperture optical receiving subsystem, the relay optical subsystem, the suboptical axis tilt detection subsystem, the beam uniformity detection subsystem, and the beam quality detection subsystem.

### 3.1. Co-Aperture Optical Receiving Subsystem Design

#### 3.1.1. Co-Aperture Optical Receiving Subsystem Design

Due to the limited size of the detector’s target surface, high-magnification beam compression was required. The co-aperture optical receiving subsystem was a diffraction-free telescope system with an optical aperture of 90 mm. While compressing the beam size, the co-aperture optical receiving subsystem must account for errors and adjustment difficulties with subsequent subsystems. Therefore, a Keplerian telescope structure, which was easier to adjust and provided a real image point, was chosen as the initial configuration. Based on the parameters of the co-aperture optical receiving subsystem design, applying the formula for the visual magnification of the Kepler telescope system and the paraxial Gaussian formula,

(3) $$\tau =-\frac{f_{1}^{\prime }}{f_{2}^{\prime }},$$

(4) $$\frac{1}{l^{\prime }}-\frac{1}{l}=\frac{1}{f^{\prime }},$$

the focal lengths of the objective group and the eyepiece group were solved to be $f_{1}^{\prime }$ and $f_{2}^{\prime }$, respectively. The calculated focal length of the objective group was $f_{1}^{\prime }=516.5\;\mathrm{mm}$, and the focal length of the eyepiece group was $f_{2}^{\prime }=86\;\mathrm{mm}$.

In the formulas, τ represents the visual magnification of the telescope system, the distance from the object point to the lens group is l, the distance from the image point to the lens group is $l^{\prime }$, and the focal length of the lens group is $f^{\prime }$. All optical surfaces in this Keplerian initial configuration were strictly coaxial. As multiple operating wavelengths need to be accommodated, chromatic aberration correction became a critical factor in improving the system’s beam quality. To eliminate chromatic aberration, the objective lens group of the co-aperture optical receiving subsystem used a combination of positive and negative lenses. The combination of flint glass and crown glass effectively compensated for chromatic aberration. By establishing an evaluation function and changing the optical glass materials, spherical aberration and chromatic aberration could be further corrected. The doublet structure could further eliminate chromatic aberration. Therefore, both the objective and eyepiece groups used single lenses combined with a doublet structure. This design not only effectively compensated for chromatic aberration but also reduced the angle of incidence by adjusting the beam height, thereby reasonably controlling and minimizing the system’s spherical aberration.

**Figure 3:**
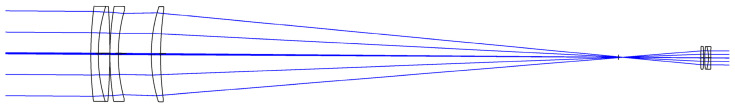
Optical diagram of common aperture receiving subsystem.

The ideal lens group from theoretical calculations was replaced with an actual lens group, and the structures of both the objective and eyepiece groups were improved. The system was optimized using optical simulation software ZEMAX V7.5.0, resulting in the final optical structure shown in Figure 3. Lens data for the co-aperture optical receiving subsystem are listed in Table 2. The lens type was standard.

#### 3.1.2. Image Quality Analysis of Co-Aperture Optical Receiving Subsystem

**Figure 4:**
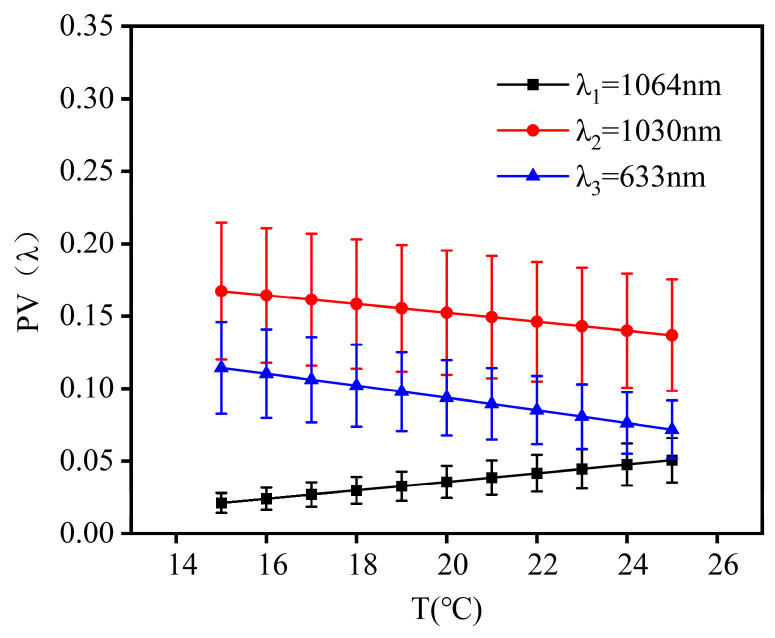
Illustration of the wave aberration of the co-aperture optical receiving subsystem as a function of temperature.

A thermal design without heat generation for the co-aperture optical receiving subsystem was implemented at 20 °C ± 5 °C. Figure 4 illustrates the wavefront aberrations of λ_1_ (1064 nm), λ_2_ (1030 nm), and λ_3_ (633 nm) laser bands at temperatures ranging from 15 °C to 25 °C. The changes in their PV values were 0.0295λ_1_, 0.0304λ_2_, and 0.0425λ_3_, while the changes in RMS values were 0.0085λ_1_, 0.00872λ_2_, and 0.0116λ_3_.

**Figure 5:**
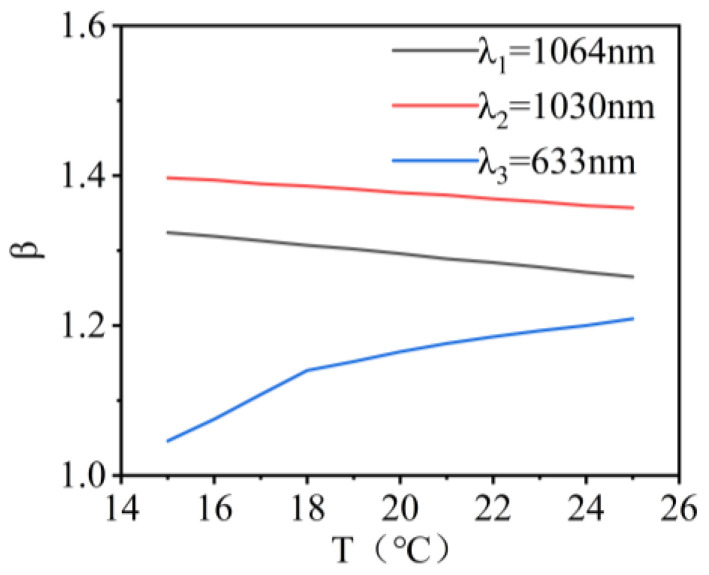
Illustration of the beam quality of the co-aperture optical receiving subsystem as a function of temperature.

The variation in beam quality for λ_1_, λ_2_, and λ_3_ wavelength lasers within the temperature range of 15 °C to 25 °C is shown in Figure 5. When the encircled energy was 83.6%, the variations in the beam quality factor β were 0.04, 0.06, and 0.16 times the diffraction limit. The results showed that the changes in the values of PV, RMS, and beam quality factor were not significant with temperature variation. This indicated that the system exhibited good imaging quality.

### 3.2. Relay Optical Subsystem Design

#### 3.2.1. Relay Optical Subsystem Design

**Figure 6:**
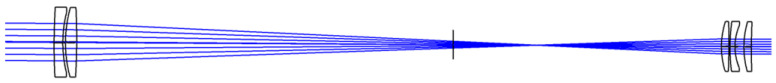
Optical diagram of relay optical subsystem.

The incident beam first passed through the co-aperture optical receiving subsystem, and after being redirected by the beam splitter and mirror, it entered the suboptical axis tilt detection subsystem, the beam uniformity detection subsystem, and the beam quality detection subsystem. Due to the limitations of the microlens size in the suboptical axis tilt detection subsystem and the detector target size in the beam uniformity detection subsystem, a relay optical subsystem was required for the secondary compression of the beam. To minimize errors arising from adjustments among the subsystems, the relay optical subsystem employed a Keplerian structure similar to that of the co-aperture optical receiving subsystem. The front group consisted of two single lenses, while the rear group employed a configuration of double cemented lenses with an additional single lens for chromatic aberration correction. The optical subsystem was repeatedly optimized using optical simulation software, resulting in a final relay optical subsystem with a beam reduction ratio of 2.499 and an exit pupil distance of 70.64 mm. The lens type was standard. Specific lens data are provided in Table 3, and the optical structure is shown in Figure 6.

#### 3.2.2. Image Quality Analysis of Relay Optical Subsystem

**Figure 7:**
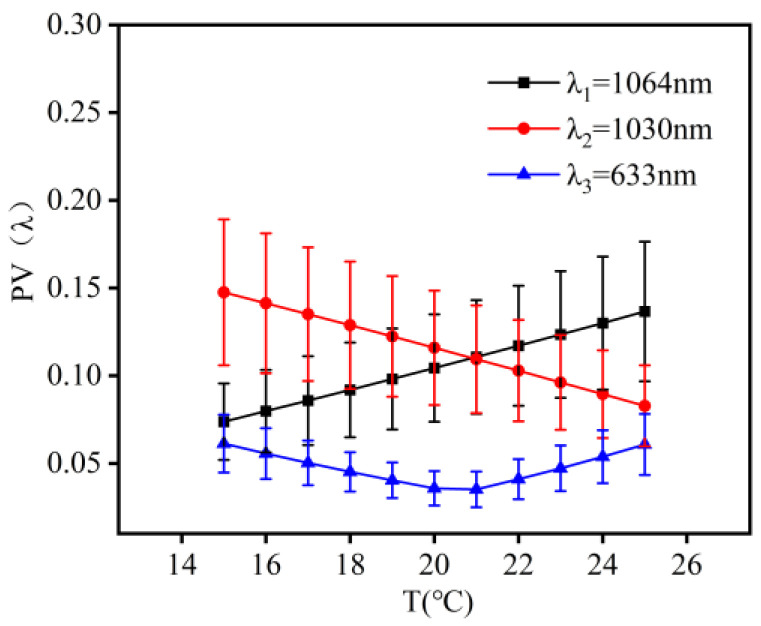
Illustration of the wave aberration of the relay optical subsystem as a function of temperature.

A thermal design without heat generation for the relay optical subsystem was implemented at 20 °C ± 5 °C. Figure 7 illustrates the wavefront aberrations of λ_1_ (1064 nm), λ_2_ (1030 nm), and λ_3_ (633 nm) laser bands at temperatures ranging from 15 °C to 25 °C. The changes in their PV values were 0.0628λ_1_, 0.0647λ_2_, and 0.0261λ_3_, while the changes in RMS values were 0.018λ_1_, 0.0185λ_2_, and 0.0076λ_3_.

**Figure 8:**
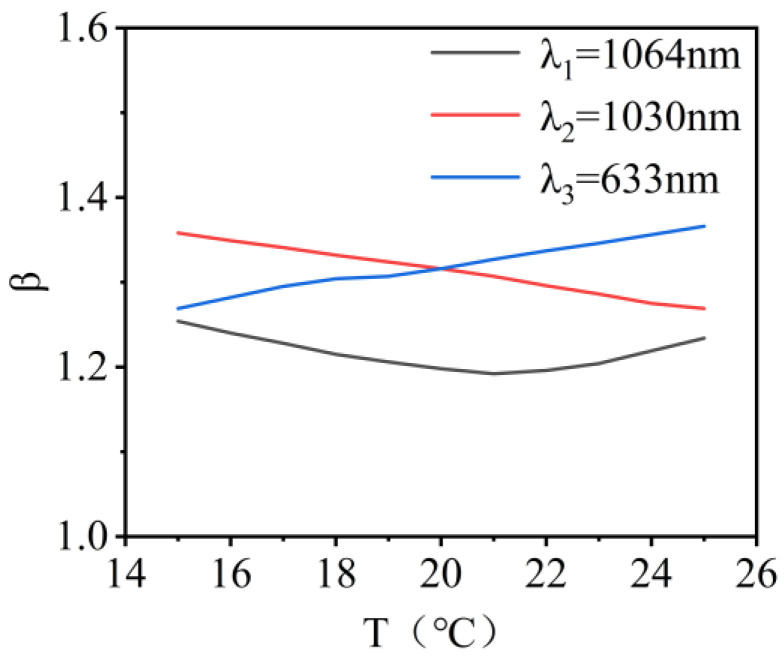
Illustration of the beam quality of the relay optical receiving subsystem as a function of temperature.

The variation in beam quality for λ_1_, λ_2_, and λ_3_ wavelength lasers within the temperature range of 15 °C to 25 °C is shown in Figure 8. When the encircled energy was 83.6%, the variations in the beam quality factor β were 0.062, 0.097, and 0.089 times the diffraction limit. This indicated that the system exhibited good imaging quality.

### 3.3. Suboptical Axis Tilt Detection Subsystem Design

**Figure 9:**
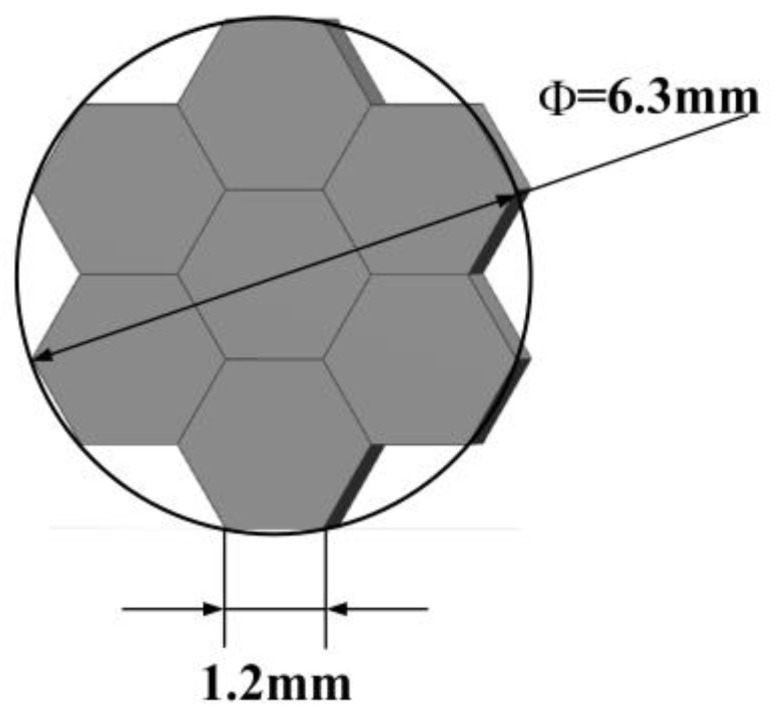
Schematic diagram of subaperture arrangement.

**Figure 10:**
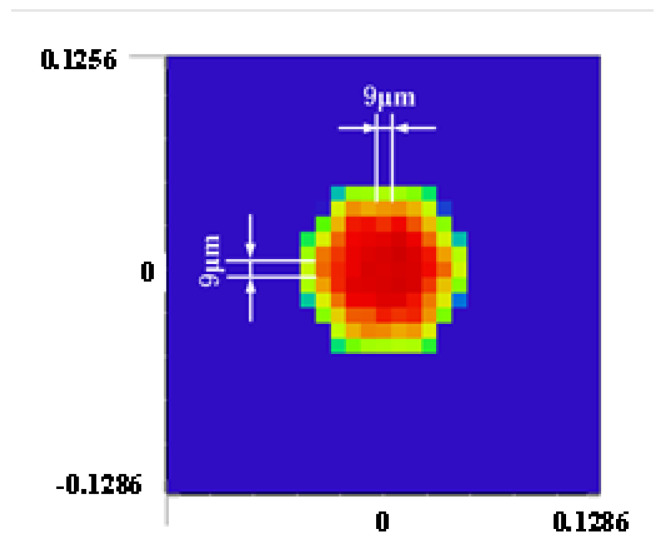
Optical simulation of microlens array.

The suboptical axis tilt detection subsystem of the coherent synthetic beam was aligned with the relay optical subsystem according to the principle of pupil matching, and the sensitivity of wavefront aberration and beam quality to temperature was consistent with that of the relay optical subsystem. Both the co-aperture optical receiving subsystem and the relay optical subsystem employed a collimator system, which compressed the beam size while establishing a conjugate relationship between the adjustment plane and the microlens segmentation of the suboptical axis tilt detection subsystem, based on the principle of conjugate imaging. A microlens array of the suboptical axis tilt detection subsystem was placed at the exit pupil of the relay optical subsystem. The subaperture parameters of the microlenses are shown in Figure 9: the edge length of the subaperture was 1.2 mm; the focal length was 100 mm; and the seven beams were arranged in a hexagonal pattern, matching the optical system’s pupil and fitting the size of the subbeam axis detector. The simulated effect of the beam after passing through the microlens array is shown in Figure 10, where the beam remained arranged in a hexagonal pattern with a pixel size of 9 μm × 9 μm, and a single beam spot contained 91 pixels. At this time, the system’s combined focal length was 1501.2 mm, which met the design requirement of a combined focal length greater than 1000 mm.

For optical systems, the quality of tolerances is one of the key factors in assessing their feasibility. Tolerances that are too strict can increase costs and make adjustments more challenging, while tolerances that are too lenient may degrade imaging quality. Therefore, the rational allocation of tolerances is crucial for optical systems. The main tolerance distribution of the system includes material tolerances, machining tolerances, and adjustment tolerances. Material tolerances primarily refer to the deviation in the refractive index of lenses, while machining errors include factors such as the thickness, tilt, eccentricity, and radius of curvature of optical components. Adjustment errors refer to the discrepancies resulting from slight positional changes of optical components due to human factors during the adjustment process.

The designed tilt phase detection system featured multiple channels and shared optical components. The tolerance analysis for systems with shared optical components generally follows this principle: all optical components should have the same tolerance values, provided they meet the imaging requirements. Based on the above principles, a tolerance analysis strategy for optical systems with shared components is proposed: prioritize the analysis of channels that are more sensitive to tolerances, and use the tolerance values of shared components in that channel as the values for the same components in other channels.

**Figure 11:**
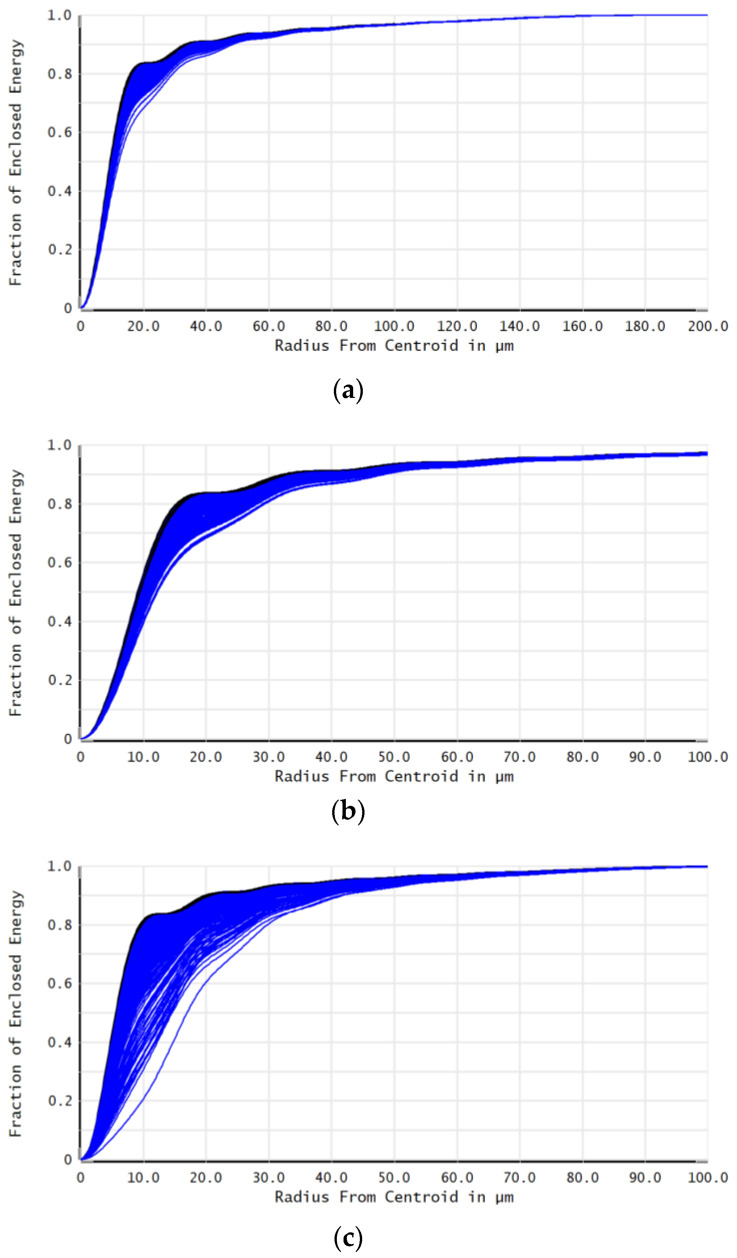
Changes in beam quality caused by machining and setting: (**a**) λ_1_, (**b**) λ_2_, and (**c**) λ_3_.

The tolerance parameters for the co-aperture optical receiving subsystem, relay optical subsystem, and suboptical axis tilt detection subsystem were set as follows: aperture of 3, thickness of ±0.05 mm, decenter X/Y of 0.03 mm, tilt X/Y of 0.025, refractive index of 0.001, and Abbe number of 0.3%. Using the Monte Carlo method, 300 computational analyses were conducted on lasers in the λ1, λ2, and λ3 bands at temperatures ranging from 15 °C to 25 °C, investigating the impacts of factors such as eccentricity and tilt on the imaging quality of the optical system. The results are presented in Figure 11: when the encircled energy was 83.6%, the beam quality β of the combined system was 1.26 times the diffraction limit in 90% of cases, satisfying the technical requirement of <1.4. At this point, it could be considered that the system’s imaging quality reached an ideal state.

### 3.4. Beam Uniformity Detection Subsystem Design

**Figure 12:**
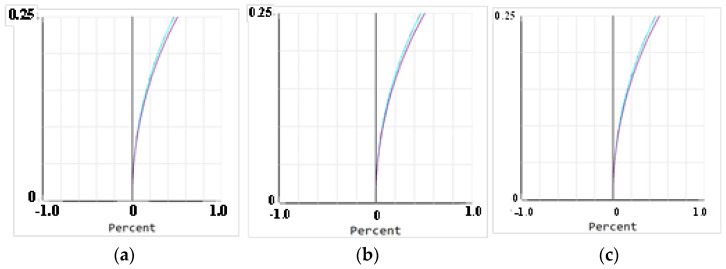
Distortion diagram of beam uniformity detection subsystem: (**a**) 15 °C, (**b**) 20 °C, and (**c**) 25 °C.

The beam uniformity detection subsystem was located at the exit pupil position of the relay optical subsystem and shared the optical path with the coherent composite beam suboptical axis tilt detection subsystem. Therefore, the wavefront aberration and the influence of temperature on the beam quality of this module were the same as those of the tilt detection subsystem and will not be repeated. When the distortion was less than 1%, the optical system could be considered to perform well. A further image quality analysis of the beam uniformity detection subsystem was conducted, and the distortion curves at 15 °C, 20 °C, and 25 °C are shown in Figure 12, with distortions of 0.4717%, 0.4720%, and 0.4277%, all of which met the requirement of distortion being less than 1%. At this point, the output spot size was 5.995 mm, which was smaller than the camera target area, allowing coverage of 667 pixels in the selected camera, thereby meeting the technical specification requirement of ≥600 pixels.

The beam uniformity detection system consisted of a co-aperture optical receiving subsystem, a relay optical subsystem, and a beam uniformity detection subsystem. The tolerance data are shown in Table 4. After conducting 300 Monte Carlo tolerance analyses, the results indicated that the RMS wavefront value had a 90% probability of being less than 0.1081λ, which met the design requirements for wave aberration.

### 3.5. Beam Quality Detection Subsystem Design

The co-aperture optical receiving subsystem and the beam quality detection subsystem were aligned according to the principles of the optical pupil. The pupil coupling principle could effectively avoid beam clipping. Under the condition that the focal length of the beam quality detection subsystem was not less than 1000 mm, the focal length of the beam quality detection subsystem was designed to be 166.85 mm. The system consisted of two plano-convex lenses and employed a retrofocus structure design. The retrofocus structure exhibited significant asymmetry and a long working distance, facilitating the installation of additional components at the rear of the system.

**Figure 13:**
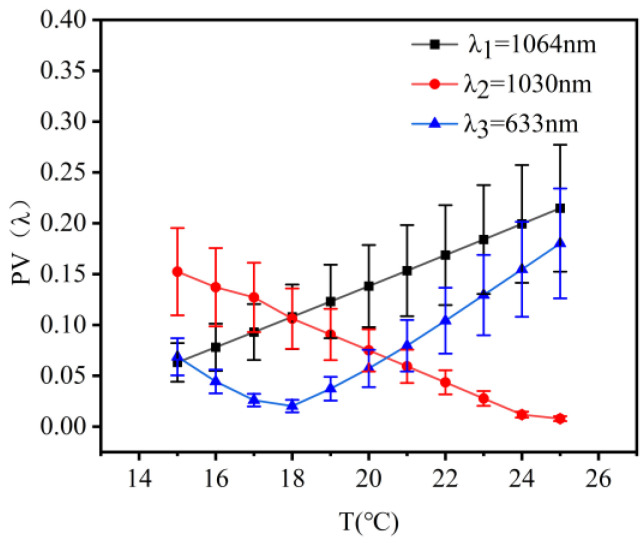
Illustration of the wave aberration of the beam quality detection subsystem as a function of temperature.

A thermal-neutral design was implemented for the beam quality detection subsystem, with a temperature range of 20 °C ± 5 °C. The wavefront aberrations of λ_1_, λ_2_, and λ_3_ band lasers in the beam quality detection subsystem are shown in Figure 13, measured at temperatures ranging from 15 °C to 25 °C. The variations in the PV values were 0.1516λ_1_, 0.1446λ_2_, and 0.16λ_3_; the variations in the RMS values were 0.0434λ_1_, 0.0405λ_2_, and 0.0479λ_3_, indicating that the system possessed stable imaging quality.

**Figure 14:**
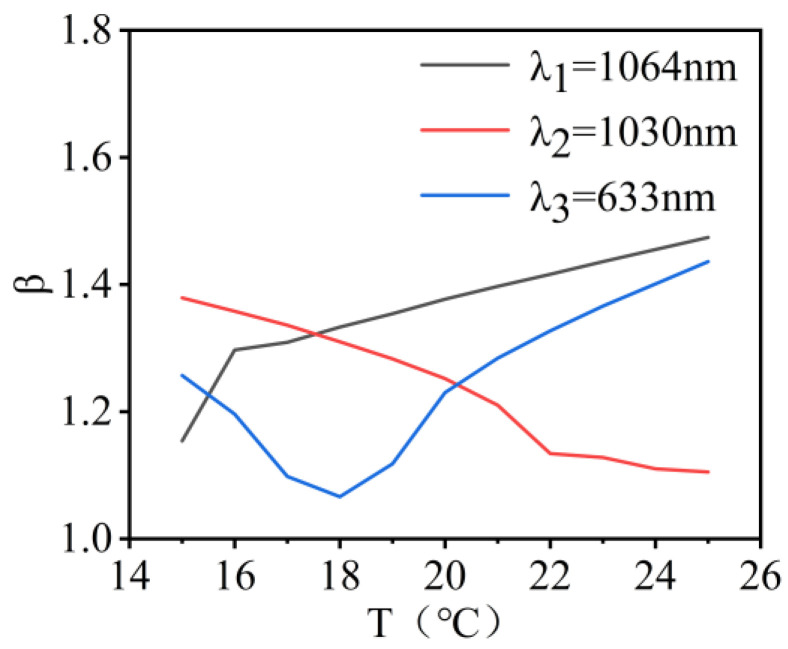
Illustration of the beam quality of the beam quality detection subsystem as a function of temperature.

Figure 14 shows the variation in beam quality of the λ_1_, λ_2_, and λ_3_ wavelength lasers within the temperature range of 15 °C to 25 °C, as detected by the beam quality detection subsystem. When the surrounding energy was at 83.6%, the variations in the beam quality factor β were 0.322, 0.374, and 0.42 times the diffraction limit, indicating that the system had a relatively stable imaging quality.

Table 5 presents the tolerance data for the beam quality detection system composed of a co-aperture optical receiving subsystem and a beam quality detection subsystem. The results of 300 Monte Carlo tolerance analyses indicated that there was a 90% probability that the RMS wavefront value was less than 0.1015λ, which met the design requirements for wavefront aberration.

## 4. Experimental Results

### 4.1. Experimental Principle

The Hartmann wavefront sensor primarily consisted of a microlens array and a CCD camera positioned at its focal plane. The microlens array at the front of the detector sampled and segmented the collimated incident beam while also segmenting the randomly distorted wavefront carried by the beam. Each microlens converged the sampled beam to form a point light spot within the corresponding window of the CCD camera. However, the distorted wavefront carried by the beam caused the light spot to shift its position within the corresponding subwindow. Before measurements are taken, calibration must be performed using an ideal light source. The measurement results indicated the deviation of the wavefront being measured relative to the reference wavefront.

#### 4.1.1. Principle of Absolute Calibration for Spherical Wavefront Using SHWFS

The optical path difference of the spherical wavefront can be expressed as a function of the radial coordinate, namely,

(5) $$\emptyset \left(r\right)=\frac{r^{2}}{2R}+\frac{r^{4}}{8R^{3}}+\ldots ,$$

R represents the curvature radius of the spherical wavefront. After passing through the microlens array, the spherical wavefront formed an array of spots on the detector, with a distance of Q  between the spots. The distance between the microlens array and the detector target surface is f, and the spacing between subapertures is h. The displacement of the spot located in the *k*-th subaperture, with a value of r=kh, relative to the center position is

(6) $$\sigma \left(r_{k}\right)=f\frac{\partial \emptyset }{\partial r}\left|r=rk\right.,$$

and taking the first-order term, we obtain

(7) $$\sigma \left(r_{k}\right)\approx \frac{khf}{R},$$

Therefore, the distance Q between adjacent spots is

(8) $$Q=\left[\left(k+1\right)h+\sigma \left(r_{k+1}\right)\right]-\left[kh+\sigma \left(r_{k}\right)\right]=h\left(1+\frac{f}{R}\right),$$

From the above equation, it can be seen that as the curvature radius R of the spherical wave increased, the distance Q between adjacent spots decreased. When R approached infinity, meaning the incident wave was a plane wave, the distance Q between adjacent spots equaled the spacing h of the microlens array subapertures. When the curvature radius ΔR of the spherical wavefront changed, the variation ΔQ in the distance between adjacent spots satisfied

(9) $$\Delta Q=\frac{hf\Delta R}{R\left(R+\Delta R\right)},$$

The initial curvature radius *R*_0_ of the spherical wavefront, when the changes in curvature were $\Delta R_{1}$ and $\Delta R_{2}$, resulted in the distances *Q*_1_ and *Q*_2_ between adjacent spots on the detector according to the equations

(10) $$Q_{1}=h\left[1+\frac{f}{R_{0}+\Delta R_{1}}\right],$$

(11) $$Q_{2}=h\left[1+\frac{f}{\left(R_{0}+\Delta R_{2}\right)}\right].$$

The changes $\Delta Q_{1}$ and $\Delta Q_{2}$ in the distances between adjacent spots on the detector are

(12) $$\Delta Q_{1}=\frac{hf\Delta R_{1}}{R_{0}\left(R_{0}+\Delta R_{1}\right)},$$

(13) $$\Delta Q_{2}=\frac{hf\Delta R_{2}}{R_{0}\left(R_{0}+\Delta R_{2}\right)},$$

the curvature radius *R*_0_ of the spherical wavefront is

(14) $$R_{0}=\frac{\left(\Delta Q_{1}-\Delta Q_{2}\right)\Delta R_{1}\Delta R_{2}}{\Delta Q_{2}\Delta R_{1}-\Delta Q_{1}\Delta R_{2}},$$

and the distance *f* between the microlens array and the detector target surface is

(15) $$f=\frac{R_{1}R_{2}\left(Q_{1}-Q_{2}\right)}{Q_{2}R_{2}-Q_{1}R_{1}}.$$

In the equation, $R_{1}=R_{0}+\Delta R_{1}$,$R_{2}=R_{0}+\Delta R_{2}$, $\Delta R_{1\;}$, and $\Delta R_{2}$ can be accurately measured. *Q*_1_ and *Q*_2_ can be calculated from the captured spherical wave point array images. According to Equation (8), the subaperture spacing *h* of the microlens array can be obtained as follows:

(16) $$h=\frac{Q_{0}R_{0}}{f+R_{0}}.$$

The calibrated sensor parameters (*f*, *h*) were incorporated into the wavefront reconstruction algorithm, using the calibrated spherical wavefront as the reference wavefront. The reconstructed wavefront represented the relative deviation of the measured wavefront from the reference spherical wavefront.

Based on the above formula for calibration, the determined physical parameters included a focal length of the lens array of 1000 mm, a center-to-center spacing of 6.2 mm for the sublenses, and a pixel size of 9 μm × 9 μm.

#### 4.1.2. A Beam Direction Detection Method Based on Far-Field Light Spots

**Figure 15:**
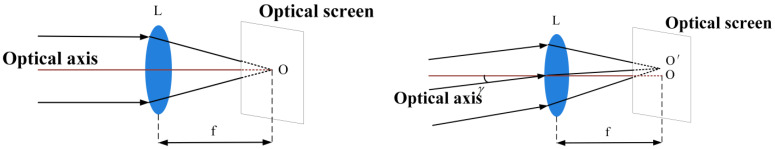
Calibration principle diagram.

Monochromatic light propagating along the optical axis, after passing perpendicularly through a convex lens, resulted in an Airy spot observed in the lens’s focal plane [19]. The center of the Airy spot was located at the intersection of the optical axis and the focal plane, with point O designated as the reference point for the beam direction at 0 μrad. When the beam exhibited wavefront distortion, the center of the Airy spot shifted. By detecting the positional shift of the light spot, the inclination of the beam could be inferred, which constituted the beam inclination detection method based on far-field light spots. The specific calibration principle is illustrated in Figure 15.

### 4.2. Experiment

**Figure 16:**
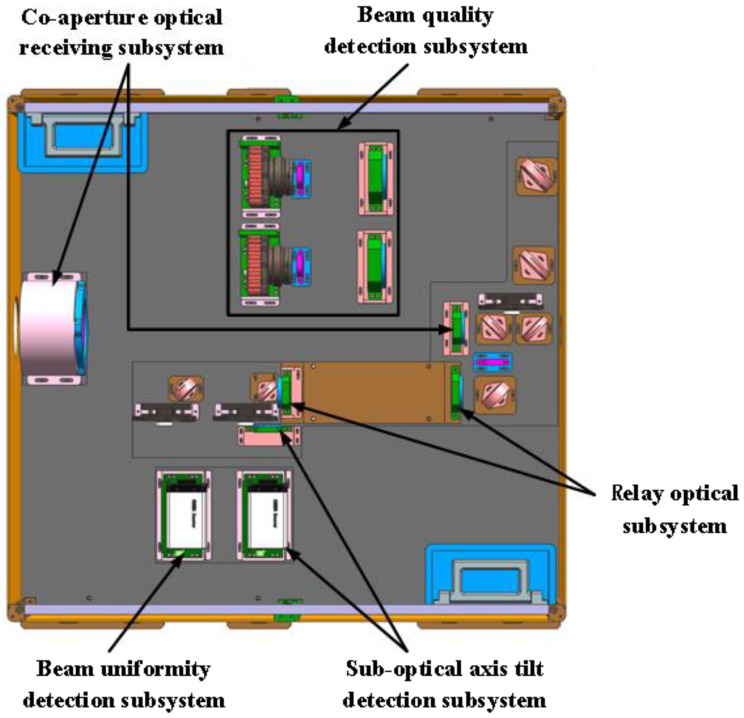
Schematic diagram of the system’s mechanical structure.

**Figure 17:**
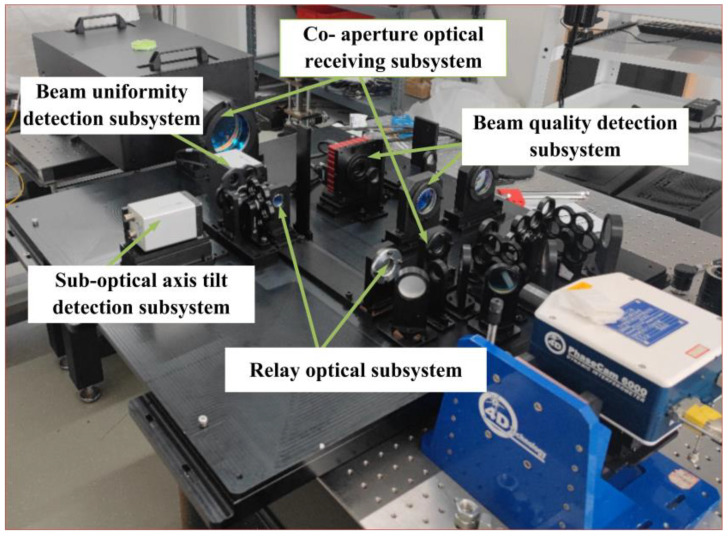
Experimental diagram of beam tilt aberration detection system.

An overall mechanical structure based on the optical system was designed, with the layout shown in Figure 16, and the experimental optical path was established. While achieving the detection of subbeam axis tilt in the coherent combined beam, the uniformity of the light spot and the quality of the beam could also be assessed. The overall experimental layout is illustrated in Figure 17.

The parallel light tube simulated a parallel light source. After the beam entered the evaluation system, it underwent beam narrowing and bending before finally reaching the various detectors. The subbeam axis tilt detection module for the coherent combined beam was used to measure the tilt of the subbeam axis, while the camera was employed to assess the uniformity of the light spot distribution and the beam quality, enabling the calculation and analysis of the beam quality β factor.

**Figure 18:**
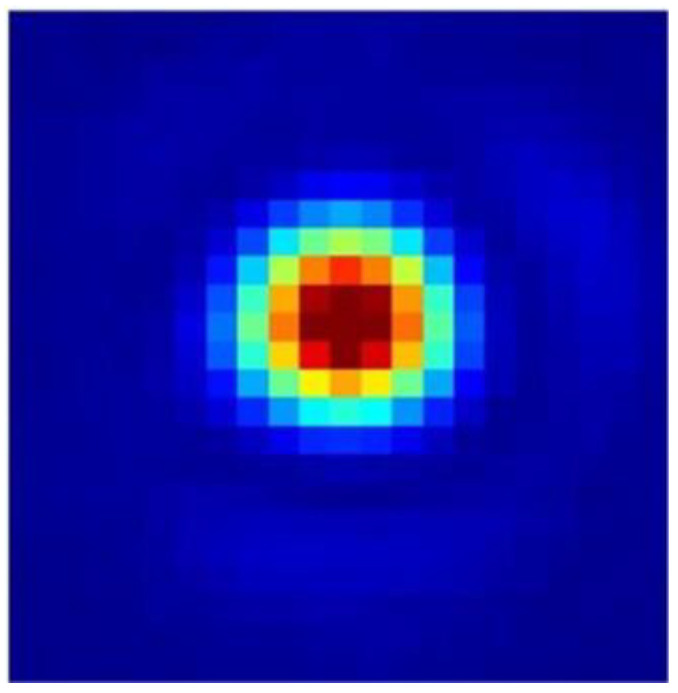
Intensity map of the sublight axis spot.

Figure 18 presents the suboptical axis spot image detected by the camera. In this study, the squared weighted centroid algorithm was chosen to process the images collected from the target surface. This algorithm used the square of the pixel intensity as the weight, which allowed for an accurate calculation of the centroid position, even if the spot shape did not closely resemble a Gaussian distribution. The camera’s pixel size was 9 μm × 9 μm, with a resolution of 1600 × 1100 pixels. Based on the atmospheric coherence length, the RMS wavefront variation for a 1 rad offset within the subregion was calculated, and the optical axis detection accuracy was determined using the equation

(17) φ=αf′.

The results indicated that the inclined detection and calibration of the seven paths of the 30 mm diameter laser could be achieved, with an inclination detection accuracy of 0.0006%.

Here, φ represents the limit resolution angle of detection accuracy, α represents the camera pixel size, and $f^{\prime }$ represents the combined focal length of the system.

**Figure 19:**
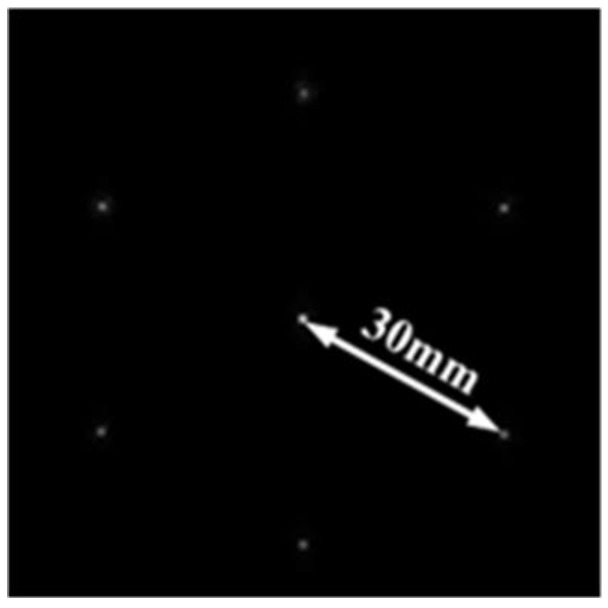
Intensity map of the seven-beam light spot.

The seven paths of the 30 mm diameter laser were compressed, split, and redirected to illuminate an array composed of seven hexagonal microlenses. This array was positioned at the exit pupil of the secondary compression, establishing an image–object conjugate relationship. The combined spots of the seven detected subpaths are shown in Figure 19.

**Figure 20:**
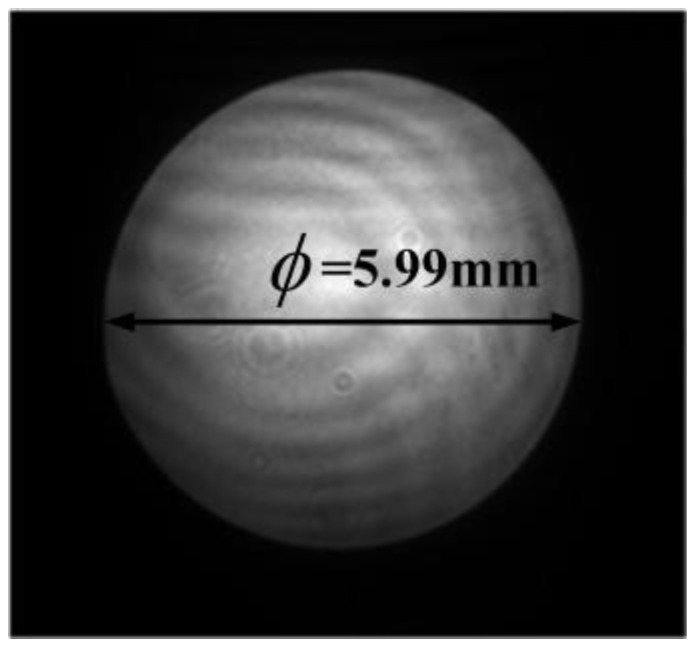
Detection image of the beam uniformity.

By detecting the phase tilt of the beam, we adjusted the incident light and examined the uniformity and quality of the corrected beam [20,21]. The image received by the beam uniformity detection camera is shown in Figure 20, with 666 pixels covering the spot image, meeting the technical requirement of covering at least 600 pixels. Using the formula

(18) $$uniformity=\left(I_{max}-I_{min}\right)/I_{ave}$$

to calculate the beam uniformity, the result indicated that the beam uniformity was greater than 0.75, meeting the specification requirement.

In the equation, *I_max_* represents the maximum intensity of the beam in the image, *I_min_* represents the minimum intensity of the beam in the image, and *I_ave_* represents the average intensity of the beam in the image.

**Figure 21:**
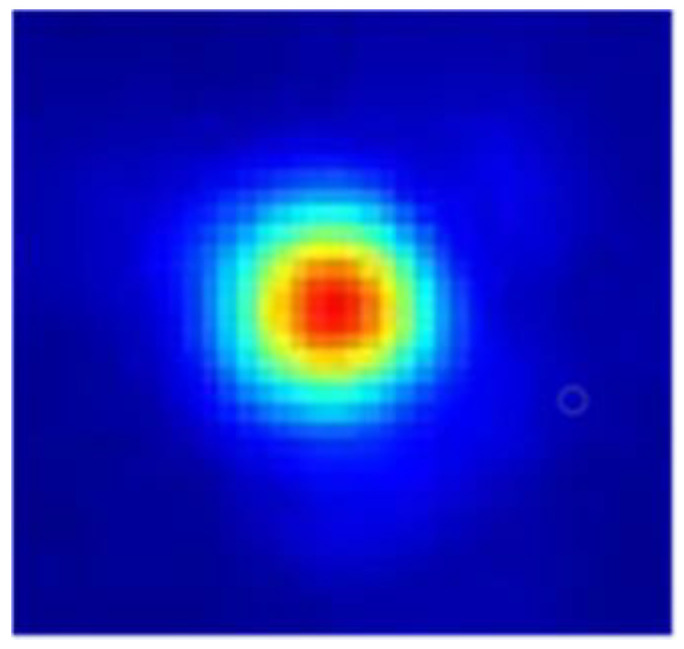
Detection image of the beam quality.

The images received by the beam quality detection camera are shown in Figure 21. In the figure, the beam quality factor *β* is superior to 1.4. The calculation involved in the beam quality factor *β* should comply with the equation

(19) $$\beta ={\left(A/A_{DL}\right)}^{1/2}.$$

Here, *A* and *A_DL_* represent the spot area of the actual system imaging when the encircling energy is 83.6% and the spot area of the system’s diffraction limit, respectively [22].

## 5. Conclusions

This paper presents the design of a coherent synthetic beam inclination detection system, which utilized a coaxial reception method in combination with a microlens array detection scheme. The system was capable of not only directly measuring the beam tilt angle but also assessing other key parameters. It allowed for the detection of optical axis inclination, as well as the evaluation of beam uniformity and quality, ultimately facilitating the synthesis of a corrected optical path. The experimental results demonstrated the system’s ability to detect the inclination of seven laser beams with diameters of 30 mm at wavelengths of 1064 ± 0.3 nm, 1030 ± 0.3 nm, and 633 ± 0.2 nm. The detection accuracy for beam inclination was 0.0006%, the beam uniformity exceeded 0.75, and the beam’s β factor was better than 1.4 times the diffraction limit. The detection accuracy of this system surpassed that of existing systems, requiring fewer iterations for optimization. After passing through the system, the incident light beam was synthesized, resulting in a high-power, high-quality beam.

In future work, the compatibility of other atmospheric window bands deserves further exploration. Meanwhile, due to the modular design used in this study, it is feasible to expand the number of arrays to increase the detection aperture. We look forward to conducting more research in the future.

## Figures and Tables

**Table 1:** The technical specifications of beam tilt detection system.

Parameter	Value
Clear aperture	90 mm
Wavelength	1064 ± 0.3 nm, 1030 ± 0.3 nm, 633 ± 0.2 nm
Field	±0.25°
The entrance pupil position of co-aperture optical receiving subsystem	500 mm
The exit pupil position of co-aperture optical receiving subsystem	≥80 mm
The multiplier of common aperture receiving subsystem	6
The combined focal length of beam quality detection system	≥1000 mm
The combined focal length of suboptical axis tilt detection subsystem	≥1000 mm
Distortion of beam uniformity detection subsystem	≤1%
The multiplier of relay optical subsystem	2.5
The number of pixels covered by beam uniformity detection subsystem	≥600
The fluctuation range of wavefront PV value and RMS value in the optical system design results.	PV ≤ λ/10, RMS ≤ λ/20
Working temperature	20 ± 5 °C

**Table 2:** The lens data of common aperture receiving subsystem.

Radius (mm)	Thickness (mm)	Glass (mm)
410.732	8.000	H-LAK7A
168.270	12.500	H-FK61B
−680.545	0.100	---
299.750	9.000	H-ZF5
172.980	35.633	---
164.454	10.000	H-LAF3B
357.310	494.231	---
∞	88.677	---
363.190	3.000	H-LAF3B
−149.806	1.263	---
111.940	4.000	H-LAK7A
−40.550	2.500	H-ZF5
671.200	50.000	---

**Table 3:** The lens data of relay optical subsystem.

Radius (mm)	Thickness (mm)	Glass
253.980	3.500	H-ZF5
50.000	1.065	---
52.062	4.500	H-LAF3B
−263.000	151.200	---
∞	107.700	---
32.909	3.000	H-LAF3B
−315.355	0.456	---
78.000	2.000	H-ZF5
21.720	3.580	---
36.086	3.000	H-FK61B
249.884	20.000	---

**Table 4:** Monte Carlo analysis results of beam uniformity detection system.

Parameter	Value			
Percentage of Monte Carlo/%	90	80	50	20
RMS wavefront	0.1081	0.0954	0.0616	0.0477

**Table 5:** Monte Carlo analysis results of beam quality detection system.

Parameter	Value			
Percentage of Monte Carlo/%	90	80	50	20
RMS wavefront	0.1015	0.0964	0.061	0.0476

## Data Availability

The original contributions presented in this study are included in the article; further inquiries can be directed to the corresponding authors.
